# Measuring similarity between gene expression profiles: a Bayesian approach

**DOI:** 10.1186/1471-2164-10-S3-S14

**Published:** 2009-12-03

**Authors:** Viet-Anh Nguyen, Pietro Lió

**Affiliations:** 1 Computer Laboratory, University of Cambridge, Cambridge, CB3 0FD, UK

## Abstract

**Background:**

Grouping genes into clusters on the basis of similarity between their expression profiles has been the main approach to predict functional modules, from which important inference or further investigation decision could be made. While the univocal determination of similarity metric is important, current practices are normally involved with Euclidean distance and Pearson correlation, of which assumptions are not likely the case for high-throughput microarray data.

**Results:**

We advocate the use of a novel metric - BayesGen - to measure similarity between gene expression profiles, and demonstrate its performance on two important applications: constructing genome-wide co-expression network, and clustering cancer human tissues into subtypes. BayesGen is formulated as the evidence ratio between two alternative hypotheses about the generating mechanism of a given pair of genes, and incorporates as prior knowledge the global characteristics of the whole dataset. Through the joint modelling of expected intensity levels and noise variances, it addresses the inherent nonlinearity and the association of noise levels across different microarray value ranges. The full Bayesian formulation also facilitates the possibility of meta-analysis.

**Conclusion:**

BayesGen allows more effective extraction of similarity information between genes from microarray expression data, which has significant effect on various inference tasks. It also provides a robust choice for other object-feature data, as illustrated through the results of the test on synthetic data.

## Background

With the development of high-throughput experimental techniques, biological research have been transformed into a data-rich discipline. DNA microarray, which allows user to measure the expression levels of thousands of gene simultaneously in a single experiment, emerged to be one of the most widely used technology. The analysis of microarray data is normally based on the reasoning that variations in gene expression patterns under different experimental conditions are the results of underlying cellular pathway changes [1]. During its inception, gene expression was analysed using semi qualitative considerations; for example, genes having the expression roughly two fold of their basal values were considered co-expressed. Nowadays we are using microarray data in a quantitative way, which has elicited the proposal of a large number of clustering algorithms tailored specifically for bioinformatics. By grouping genes with similar expression profiles into clusters, researchers have been able to make meaningful inference of regulatory modules and functional pathways [2].

Before a clustering procedure could be performed, it is naturally conceivable to ask if the metric of similarity between expression profiles has been univocally determined. While the normal practice has been largely involved with Euclidean distance and Pearson correlation, these metrics either assume a clean experimental space or the linearity between similar genes, which are not likely the case for high-throughput expression data. We expect a metric that could handle the dependency on the responsiveness/determination accuracy of different concentrations, and the nonlinearities that are likely to result from the production of mRNAs and deficiencies of measuring devices. Moreover, as commonly observed, the measurement average and dependency may be linked, or high intensity values are likely to be affected by larger error [3].

In this paper we apply Bayesian model selection to construct a principled framework for similarity/distance definition. One emergent feature about Bayesian approach is that it requires explicit statement of underlying assumptions, making it easier for users to evaluate the suitability of a given metric. We then propose a novel distance metric that addresses the nonlinearity and the variation of noise levels across different microarray value ranges, through the joint modelling of data points' intensity levels and noise variances. Another important aspect is that by deriving a full Bayesian model, it also facilitates the employment of meta-analysis through the estimation of the hyper-parameters.

### Bayesian model selection

Bayesian model selection uses the probability rules and Bayes theorem to choose among alternative hypotheses. To evaluate the plausibility of a given model *H*, one considers the probability of the data *D *given *H *(or the evidence of *H*) by marginalising over unknown parameters:

This quantity automatically encodes the Occam's factor or the preference for models with more constrained generating mechanism. In other words, since complex models have the capability of explaining over a wider range of data, their evidence distributions are more widely spread over the data space. Hence, if *H*_2 _is a more complex model compared to *H*_1_, and given a data *D *that could be explained by both *H*_1 _and *H*_2_, the evidence *P *(*D|H*_1_) will be larger than *P *(*D|H*_2_). More detailed discussion about Bayesian model selection could be found elsewhere [4].

Bayesian evidence serves as the basis for Bayes Factor (BF), which is defined as the evidence ratio *P*(*D|H*_1_)/*P *(*D|H*_2_) times the hypotheses prior ratio *P *(*H*_1_)/*P *(*H*_2_). In the case of no prior bias exists between the two hypotheses, the prior ratio could be safely ignored. Due to the Bayesian evidence's ability of automatically choosing the right model, BF was known to be more robust with sparse data in comparison to the popular likelihood ratio test (LRT). However, this advantage comes at the computational cost of integration over the parameter space, which normally employed Monte Carlo intergration and importance sampling [4]. Another option is to estimated BF through asymptotic approximation methods such as Bayesian information criterion (BIC) [5].

Bayes Factor and its approximated versions have recently attracted more interest as a tool for selecting alternative hypotheses in bioinformatics [6-8]. The task of grouping similar gene expression profiles into clusters was also recently formulated under the Bayesian framework [9]. Although considering a full Bayesian generative model, the authors assigned genes to clusters by estimating the single point at which the posterior distribution over latent variables was maximised. This MAP (maximum a posterior) approach inherently used Euclidean distance to measure expression profile similarities.

In this paper, we apply the model averaging principle of Bayesian evidence, which takes into account all possible models rather than relying on the best one. We start by constructing the general Bayesian formulation for pairwise similarity/distance measurement, from which the new distance metric BayesGen is described. We then compare BayesGen performance with Euclidean distance and Pearson correlation through three test sets. The first test on simulated data suggests that the full Bayesian approach is better in differentiating homologous and heterogeneous pairs. The second test on two genome-wide *S. cerevisiae *datasets examines the capability of similarity/distance metrics in directly inferring the pairs of interacting proteins. The last test on four human cancer datasets quantifies the effect of metric selection on hierarchical clustering results, both in terms of cluster structure and partition accuracy. BayesGen delivers best or competitive performance in all cases.

## Results and discussion

### Bayesian pairwise distance

Suppose we are interested in a set of *n *objects {**o**_1_, ..., **o**_*n*_} of which observations of their behaviour are available as dataset *D *= {**x**_1_, ..., **x**_*n*_}. We assume an object behaviour is the result of an underlying generative process that takes into account its properties. We formalise such generative process as a probability distribution *F*(.|***θ***) over the experimental space, where all observation vectors are generated from.

The similarity between two objects **o**_*i *_and **o**_*j *_would be best specified as the similarity between their inherent properties. Although such information is not directly available to us, it has been encoded into the generative processes that resulted in our observations. The similarity between *o*_*i *_and *o*_*j *_could then be defined to be proportional to the probability that two samples **x**_*i *_and **x**_*j *_were generated from the same process. Denoting *H*_*same *_as the hypothesis that **x**_*i *_and **x**_*j *_are from a single process, and *H*_*diff *_as its complement (two samples were generated from two different processes), we have:

where *s*(*i*, *j*) is the similarity function between two objects **o**_*i *_and **o**_*j*_, and *p*(*H*_*same*_) and *p*(*H*_*diff*_) are the prior beliefs. Since similarity/distance measurement are invariant to monotonic transformations, we could define a distance measurement between two objects **o**_*i *_and **o**_*j *_as the Bayes factor between the two hypotheses, employing the evidence expansion from (1):

#### BayesGen distance for gene expression data

Given a dataset *D *measuring the expression of *n *genes through *d *different experimental conditions, our objects of interest could be the set of *n *genes or the set of *d *conditions. Without violation to generality, we assume a default interest on genes.

As cellular processes are carried out through the coordination of gene modules, where the expression levels of co-regulated genes within each module are similar under a given condition, we assume that each sample **x**_*i*_, *i *= 1.. *n *is generated from a Gaussian distribution with parameter ***θ ***= {***μ***, Σ}. ***μ ***is a vector of length *k *that defines the expected expression level under *k *conditions, while Σ specifies the variance range accounted for measurement noise and cellular process inherent stochasticity. The formal generative process for each experimental condition is as follows (we remove parameters' sub- and super-scripts for presentation clarity):

where ***μ***_0_, Σ_0_, *λ*_0_, and *ν*_0 _are hyperparameters indicating the prior mean, prior variance, and their belief levels respectively. Note that ***μ ***and Σ are not independent, reflecting the dependency between variance and intensity levels observed in expression data. The generative process has two stages: firstly, different processes are generated by mutating the global mean ***μ***_0 _with the expected variance of Σ_0_; secondly, each process's samples are generated by adding fluctuations Σ to its expected expression level ***μ***. Suppose that all observations of the given condition could be fitted to a Gaussian distribution  (**m**, **V**), our model hyper-parameters should be estimated such that:

Assuming no prior knowledge, the expected decomposition of process-generated and sample-generated variance is equiprobable and equals **V**/2. Plugging the model of (6-8) to (5), and assuming that Σ is a diagonal matrix, we obtain the closed-form formula for BayesGen distance measurement for two given genes *i *and *j *as follows:

where

and **m**^*k*^, **v**^*k*^, , and  are the *k*th component of the data global mean and variance, and the two sample local mean and variance respectively.

### Experiment 1: Synthetic data

The first experiment was designed to compare the capability of the three metrics in differentiating between sample pairs that are generated from a single process, and those generated from two different processes. In order to explore the strengths and weaknesses of the metrics in a reasonably exhaustive way, we use synthetic data with different generating assumptions, which are not necessarily the valid assumptions for real microarray expression datasets.

We conducted the test over three cases, distinguished by the way samples within a process are linked: (1) Samples are independently generated from a Gaussian distribution, with different expected noise levels for different conditions; (2) Samples are independently generated from a Gaussian distribution, with fixed noise levels over all conditions; (3) Samples are generated as linear transformations from a common mean vector, with random noises added.

A dataset is the composition of 200 samples coming from two different processes (100 samples each). The distances between all pairs in the dataset were calculated, ranked, and scaled so that they are evenly distributed over the range [0, 1]. We then grouped distance values into two classes by the origin of their objects: *within *(the two samples were from the same process), and *across *(the two samples were from different processes). The results are averaged over 100 independent datasets for each case.

**Figure 1:**
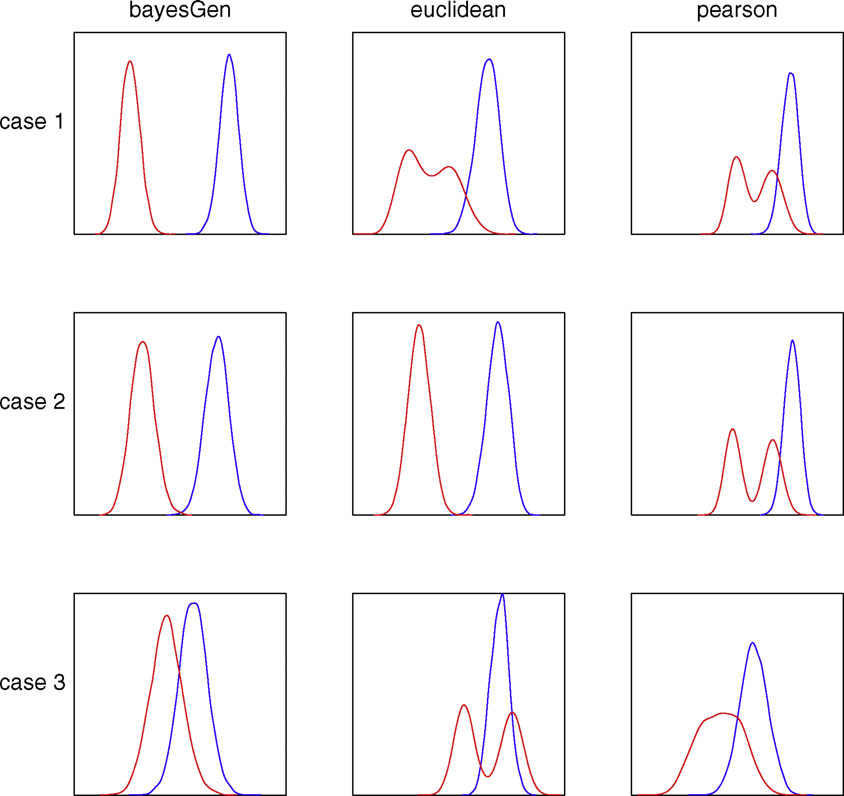
**Distance distributions of the homologous and heterogeneous groups**. Comparison of the three distance metric capability in differentiating between homologous and heterogeneous sample pairs over three generating cases. Red lines: densities of homologous distances (two samples are from the same process); blue lines: densities of heterogeneous distances (two samples are from two different processes). Case 1: Samples are independently generated from a Gaussian distribution with varying noises (favours BayesGen); Case 2: Samples are independently generated from a Gaussian distribution with fixed noise (favours Euclidean distance); Case 3: Samples are generated as noisy linear transformations from a common mean vector (favours Pearson correlation).

Figure 1 shows the distance distributions of the two classes (red to *within*, blue to *across*) obtained when BayesGen, Euclidean distance, and Pearson correlation were used as distance metrics over the three cases. The intersection between the two lines could be interpreted as the probability of error when using the distance as a tool for sample origin prediction. As expected, each metric was the best choice under its favoured assumptions. However, while Euclidean distance and Pearson correlation performance deviated when getting off their favouring case, BayesGen remained to be the best or competitive to the best over all three cases, suggesting its position as the safe choice for most application problems.

### Experiment 2: Functional association discovery

In the second experiment, we examined the direct application of the proposed measurement approach in predicting protein pairs that participate in the same cellular processes from high throughput microarray expression data. Our application was based on the guilt-by-association heuristic [1], which says that genes with similar expression profiles are likely to belong to the same functional module. Using this heuristics, co-expression gene networks were often constructed by Pearson correlations for all gene pairs [10].

#### Datasets

We used two public datasets measured genome-wide gene expressions of *Saccharomyces cerevisiae *under different experimental conditions. Each row corresponds to a gene, which we treated as a sample, and each column corresponds to a sample feature.

The first dataset was extracted from the gene expressions of wild-type and Mec1 defective yeasts in response to two different DNA-damaging agents: methylmethane sulfonate and ionising radiation [11], making a total of 52 observed features for each gene. The experiments were performed on spotted microarrays.

The second dataset contains the gene expressions from triple replicates of 14 yeast samples differentiated by their sucrose gradients [12], making a total of 42 features for each gene. The experiments were also performed on spotted microarrays, with the focus on protein biosynthesis process.

Since the purpose of our experiment was to evaluate the proposed measurement directly, without intervention from any other algorithms, we did not apply any imputation method here. All the rows that contain missing values were ignored, leaving a total of 2,222 genes for [11] and 1,758 genes for [12]. To account for possible unfairness towards traditional approaches due to the inherent column-wise normalisation of BayesGen, we created a normalised version for each dataset, on which later we repeated the tests for Euclidean distance and Pearson correlation. Formally, for each column *X*^*k*^, the following transformation was applied:

where  and Σ^*k *^are the mean and variance of feature *k*, leaving each feature column with the mean of 0 and variance of 1.

#### Experiment and results

For each dataset, 5 pairwise distance matrices were computed using: Euclidean distance on original data, Euclidean distance on normalised data, Pearson correlation on original data, Pearson correlation on normalised data, and BayesGen on original data (BayesGen has the inherent column-wise normalisation in its formula).

Given a distance matrix, the smallest *t*% were marked as positive pairs, which means protein pairs that belong to the same molecular process, where *t *is a user-specified threshold. For our experiment, we ranged *t *from 0.01 to 7.

**Figure 2:**
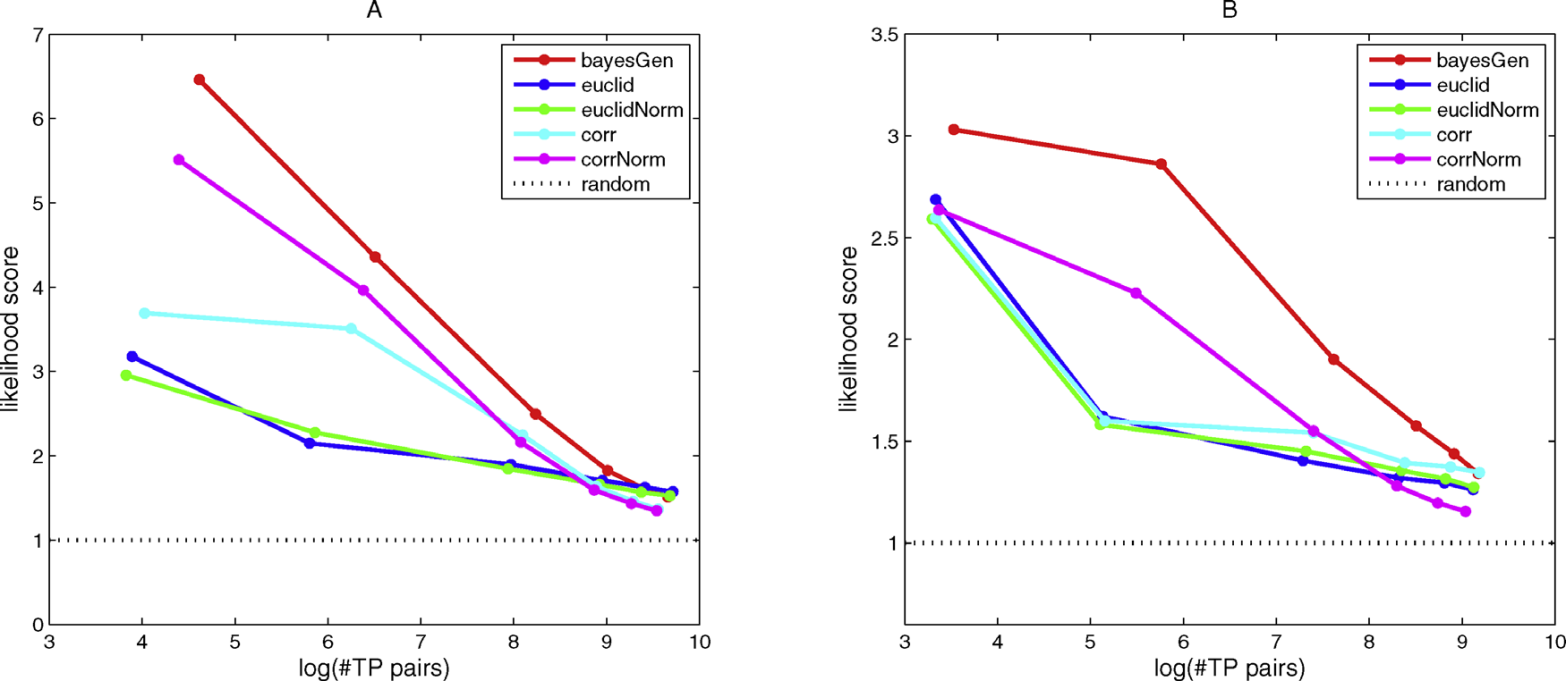
**Protein functional association discovery**. Comparison of the three distance metric capability in predicting interacting yeast protein pairs from genome-wide microarray expression data. The standard positive pairs are derived from the annotations of GO terms that got 5/6 votes of expert survey. (A) Results from Gasch et al. [11] data; (B) Results from Avara et al. [12] data.

To evaluate the quality of our prediction, we compared the predicted pairs against the positive pairs derived from the combination of Gene Ontology (GO) [13] and the associated annotations of *S. cerevisiae *[14]. Both the GO term and annotations files were downloaded from [15] on 16/02/2009. Since the GO structure consists of several thousands of terms, each of different levels of specificity, counting any protein pairs that were co-annotated by a GO term as positive would be misleading. We selected a list of 140 qualified terms which got 5/6 votes in the survey performed by Myers et al. [16] on the validity of GO terms for concluding that co-annotated proteins actually interact. We obtained 2,467,531 pairs for the 2,222 genes presented in Gasch et al. [11] data, and 1,544,403 pairs for the 1,758 genes of Avara et al. [12] data, which are equivalent to 6.6% and 7.2% of all possible pairs respectively, and agreed with estimated proportion of yeast (about 5%). The results are shown in Figure 2.

### Experiment 3: Hierarchical clustering application

The aim of the third experiment was to quantify the advantage of the proposed approach in application to a distance-based clustering method. We chose agglomerative hierarchical clustering due to its popularity in the area of gene expression analysis. Starting from a set of *N *objects, considered as *N *clusters, the algorithm iteratively builds up a tree by linking the two closest clusters at each step. It goes through *N - *1 steps in total, resulting in a single tree for all the objects.

#### Datasets

We used four public datasets of gene expression profiles measured on cancer patients during the diagnosis stage [17]. Unlike the previous experiment, here we treated patients as the objects of interest, and genes as features. The classification of patients into distinct classes was known a priori, and only used for evaluation purpose.

The first dataset contained bone marrow samples obtained from acute leukemia patients, measured on the Human Genome HU6800 Affymetrix microarray [18]. Among the 38 patients, 11 were of acute myeloid leukemia (AML), and 27 were of acute lymphoblastic leukemia (ALL). The ALL group could be further divided into 2 subtypes: T-lineage (8 samples), and B-lineage (19 samples), making a total of 3 known classes.

The second dataset consisted of leukemia bone marrow samples from ALL-type pediatric patients, measured on the Human Genome U95 Affymetrix microarray, with the focus on the patients' risk of relapse [19]. Among the 248 samples, 43 were of T-lineage, and 205 were of B-lineage. The B-lineage groups was further divided into 5 prognostically important subtypes: 15 containing t(9;22) [BCR-ABL], 27 containing t(1;19) [E2A-PBX1], 79 containing t(12;21) [TEL-AML1], 20 containing rearrangements in the MLL gene, and 64 containing hyperdiploid karyotype, making a total of 6 known classes.

The third dataset contained 103 cancer samples from 4 distinct tissues (26 breast, 26 prostate, 28 lung, and 23 colon), measured on the Human Genome U95 Affymetrix microarray [20].

The last dataset consisted of diagnostic samples from diffuse large B-cell lymphoma patients, measured on the Human Genome U133A and U133B Affymetrix microarrays [21]. Among the 141 subtypes, 3 discrete subtypes had been identified: oxidative phosphorylation (49 samples), B-cell receptor/proliferation (50 samples), and host response (42 samples).

Since it is possible that the datasets contained multiple signatures other than the known phenotypes, they had been preprocessed by applying a signal-to-noise ratio test and selecting the most up-regulated genes for each class [22], so that the observed phenotype would be the dominant signature in the data.

#### Experiment and results

For each of the described dataset, we calculated the distance matrix using the 5 approaches: Euclidean distance, Euclidean distance with z-score normalisation, Pearson correlation, Pearson correlation with z-score normalisation, and the newly proposed BayesGen. These distance matrices were then fed as inputs to the agglomerative hierarchical clustering to obtain one linkage tree for each metric. We used average linkage, which defines the distance between two clusters as the average of all between-cluster distances. Formally, given 2 clusters *C*_1 _and *C*_2 _of *n*_1 _and *n*_2 _objects respectively, the distance between *C*_1 _and *C*_2 _is:

Hierarchical clustering does not require users to specify the number of clusters beforehand. One could later decides on the number of partitions by looking at the tree structure. However, this process is normally bias and based on one's prior expectation about the data. In an attempt of achieving a reasonable fairness level for all approaches, we estimated the appropriate number of clusters for each tree using gap statistics [23]. The idea of gap statistics is to find the point at which the within-cluster dispersion is minimised, by comparing it to a null reference distribution. More details about gap statistics is in the Method section. To evaluate the predicted clusters quality we used the adjusted Rand index [24] to compare between the known class labels and the cluster labels. The index ranges from 0 to 1, where 1 corresponds to perfect agreement, and 0 to the expected value of random cluster assignment. The computation detail of Rand index goes in the Method section.

Table 1 presents the adjusted Rand indices obtained using different distance matrices as the input for the hierarchical clustering and gap statistics procedure. While the Bayesian generative input is the clear winner for all 4 data sets, the position of the second best fluctuates among normalised Pearson correlation, Pearson correlation, and normalised Euclidean distance. Although z-score normalisation overally increased the performance of both Euclidean distance and Pearson correlation, in some cases it may lead to the loss of necessary information and decreased clustering accuracy.

**Table 1:** Clustering expression profiles into cancer subtypes

	euclid	euclidNorm	corr	corrNorm	bayesGen
General leukemia	0.5447	0.1175	0.7491	0.1817	0.8076
Pediatric leukemia	0.1982	0.4789	0.2014	0.9129	0.9413
Multiple tissues	0.5304	0.9082	0.6416	0.783	0.9726
B-cell lymphoma	0.0016	0.0008	0.4407	0.1745	0.9053

Average	0.3187	0.3764	0.5082	0.5130	0.9067

**Figure 3:**
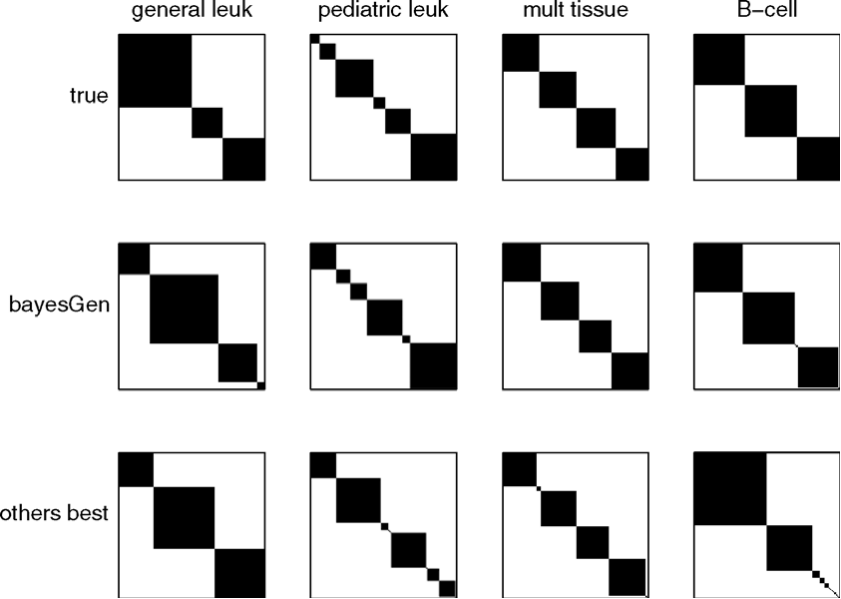
**Cluster structures resulted from the use of different metrics on hierarchical clustering**. Comparison of the resulted cluster structures resulted from the use of different distance metrics on hierarchical clustering over 4 cancer datasets. Top row: the true structure derived from known phenotypes; Middle row: the structure resulted from BayesGen (offered highest Rand indices); Bottom row: the structure resulted from the metric that offered the second best Rand indices.

The numbers of clusters estimated from gap statistics are shown in table 2, and the cluster structure obtained from hierarchical clustering partitions are in figure 3. For clarity, we only show the structures of the best (which is always Bayesian generative), and the second best partitions (as of table 1) for each dataset. It can be seen that both in terms of cluster number estimation, and cluster structure, our approach outperformed the remaining four.

**Table 2:** Predicting number of clusters using gap statistics

	true number	euclid	euclidNorm	corr	corrNorm	bayesGen
General leukemia	3	3	3	3	3	4
Pediatric leukemia	6	3	13	2	15	7
Multiple tissues	4	6	7	6	9	4
B-cell lymphoma	3	2	2	15	15	6

Average difference		1.2	2.2	3.6	5.2	1.0

## Conclusion

We suggested the use of BayesGen - a new metric for measuring similarity/distance between gene expression profiles. Based on the observation that both data points' intensity levels and their relative variance jointly contribute to the identification of the underlying cellular processes, the metric was derived using a full Bayesian approach, which incorporates as prior knowledge the global characteristics of the whole dataset.

In comparison to Euclidean distance and Pearson correlation, BayesGen was shown to be the superior in predicting the interacting protein pairs through the construction of pairwise relevance networks. The profound effect of metric selection on clustering results was confirmed in the last experiment, showing significant improvement brought by BayesGen to hierarchical clustering both in term of partition accuracy and cluster structure. Although encoding more information, BayesGen shares the calculation simplicity of the other two, and we expect its seamless integration capability to any downstream distance-based approach.

Despite the inspiration from gene expression data, BayesGen was designed with a general purpose in mind, and could be well applied to other object-feature data. The test on synthetic data under different generating assumptions showed that BayesGen is robust enough to be considered as the safe choice in most cases. Work in progress is to extend the same Bayesian framework to other data types, including relational and structured data.

## Methods

### Euclidean distance

The Euclidean distance between two expression profiles **x**_*i *_and **x**_*j *_is defined as follows:

which measures the absolute distance between expression profiles in the *d*-dimensional experimental space. From the statistical point of view, the unification between Bayesian distance and Euclidean distances occurs when generative processes are differentiated by only their expected intensity levels or ***θ ***= {***μ***}, and no restriction is put on the selection of possible means. Formally, each condition data points are generated as follows:

where  is a constant and ℛ is the set of all real numbers. Plugging this model to (5), and assuming that Σ_0 _is of the form *λ I*, the resulted distance between two given genes *i *and *j *is:

where

which is the squared version of Euclidean distance.

### Pearson correlation

The Pearson correlation between two expression profiles **x**_*i *_and **x**_*j *_is defined as follows:

where  and ) are the average expression levels over all *d *experimental conditions of gene *i *and gene *j *respectively. Pearson correlation takes lightly the magnitude of each data point, but pays attention to the shapes of vectors. It ranges from -1 to +1, with the absolute value specifies the level of correlation between two variables, and the sign indicates the direction of such correlation. The perfect correlation *corr*(*i*, *j*) = 1 is reached when **x**_*i *_= *a***x**_*j *_where *a *is a random coefficient. Using Pearson correlation as a tool of similarity/distance measurement, one may choose to pay attention only to positive correlations, or utilise also the negative values by taking their absolute values.

Note that while Euclidean and Bayesian distance treat each expression profile **x**_*i *_as a vector of *d *features, Pearson correlation treats it as a sequence of *d *independent observations of object *i*.

### Gap statistic

Gap statistic [23] was developed to estimate the appropriate number of clusters  for a dataset given the set of partitions resulted from a clustering technique with *k *= 1 .. *K*. Although its specific computation was designed for k-means clustering, gap statistic is applicable to any method of data groupings. To estimate the number of clusters from a linkage tree, we applied gap-uniform with empirical adjustment in the last step of deciding the appropriate .

Given a partition with *k *clusters *C*_1_, ..., *C*_*k *_of size *n*_1_, ..., *n*_*k*_, the dispersion index at *k *is defined as:

where *D*_*r *_is the sum of within-cluster distances of cluster *r*:

At each point of *k*, gap statistic compares this dispersion index with its expectation under the null hypothesis. The null hypothesis assumes that the whole dataset is of a single uniformly distributed cluster. The expectation  is computed by averaging over *B *randomly generated datasets. The gap statistic is defined as follows:

where

Tibshirani et al. [23] suggested to choose the number of clusters as the smallest *k *such that

where *sd*_*k*+1 _is the standard deviation of  over *B *simulated datasets. However, in the case of hierarchical clustering, where the resulted trees may contain leafs or very small clusters, we suggest that *k *should be estimated as follows:

1. Choose candidate numbers {*k*^*c*^}as all *k *that satisfies *G*_*k *_≥ *G*_*k*+1_.

2. Choose the number of clusters as the smallest *k*^*c *^such that .

### Adjusted Rand index

The Rand index [25] was developed to measure the agreement of two partitions of the same set of *N *objects. The two key assumptions underlying its derivation are: first, each object is assigned to exactly one cluster; and second, all objects are of equal importance in forming the clusters. Given two partitions *P*_1 _and *P*_2_, with *k*_1 _and *k*_2 _groups respectively (*k*_1 _and *k*_2 _are not necessarily equal). The matching of the two partitions is defined as confusion matrix **C **of size *k*_1 _× *k*_2_, where *C*_*ij *_is the number of objects in group *i *of partition *P*_1 _that are also in group *j *of partition *P*_2_. Rand index computes the probability that any 2 out of *N *objects were grouped similarly. However, it is not well scaled and is bias towards increasing number of clusters.

The adjusted Rand index [24] addressed these issues, and is computed as follows:

where

and

## Competing interests

The authors declare that they have no competing interests.

## Authors' contributions

VAN conceived and designed the study, carried out experiments, and drafted the manuscript. PL supervised the work, discussed the results and critically revised the paper. Both authors read and approved the final manuscript.

## Note

Other papers from the meeting have been published as part of *BMC Bioinformatics* Volume 10 Supplement 15, 2009: Eighth International Conference on Bioinformatics (InCoB2009): Bioinformatics, available online at http://www.biomedcentral.com/1471-2105/10?issue=S15.
